# Supplementary material for: Are carbon emissions trading and green financial instruments synergistic? -Comprehensive quantitative research based on content analysis
Source: PLoS One. 2024 Mar 7;19(3):e0298601. doi: 10.1371/journal.pone.0298601 (PMC10919601; doi:10.1371/journal.pone.0298601)
Supplement: S1 Data — (ZIP) [file pone.0298601.s001.zip › Data/地级市Z_F工作报告.zip!/╝¬┴╓/░╫╔╜╩╨/└┤╫╘╣1⁄2╓┌║┼í╛┬φ┐╦╩2╛▌═°í┐_2008.docx]

各位代表：

　　我受市政府委托，向大会报告工作，请予审议，并请各位政协委员提出意见和建议。

　　一、回顾过去三年，白山实现历史新跨越

　　2005年以来，我们在省委、省政府和市委的领导下，团结带领全市各族人民，以科学发展观为统领，以富民强市为目标，紧紧抓住振兴东北老工业基地和资源型城市经济转型试点市等战略机遇，开拓创新，扎实苦干，开创了前所未有的新局面。这三年，是白山历史上发展速度最快、发展质量最好、城乡面貌变化最大、人民得到实惠最多、改革开放成果最为显著的三年。

　　综合实力明显增强，三年实现了“四个新突破”。经过全市上下共同努力，白山经济步入了更好更快的发展时期。经济总量实现新突破。预计2007年实现地区生产总值235亿元，比2004年增长78.3%；人均GDP达到1.8万元，比2004年高出8300多元。三年累计实现地区生产总值587亿元，是前五年的1.3倍。财政收支实现新突破。预计2007年完成全口径财政收入20亿元、地方级财政收入10亿元，分别比2004年增长1.2倍和1.4倍，增幅连续三年居全省前三位。财政支出40亿元，比2004年翻了一番，人均支出居全省前三位。固定资产投资实现新突破。预计2007年完成全社会固定资产投资200亿元，比2004年翻了两番多。三年累计完成固定资产投资406亿元，是前五年的2.3倍。建成千万元以上项目274个，其中亿元以上项目83个，是投资额度最大、新上项目最多的三年。产业结构调整实现新突破。预计第一产业实现增加值31亿元，比2004年增长40%。第二产业实现增加值131亿元，比2004年翻了一番多。第三产业实现增加值73亿元，比2004年增长64.9%。三次产业比重由2004年的18.2:52.8:29调整到13.2:55.7:31.1。

　　改革开放纵深推进，三年取得了“四个新成就”。紧紧抓住改革开放不放松，着力推进体制机制创新，经济发展的动力和活力不断增强。争取政策空间取得新成就。经过坚持不懈地努力，我市被国家确定为综合资源型城市经济转型试点市和可持续发展实验区，被命名为中国特产名城，争取国家循环经济试点市工作全面启动，赢得了更为广阔的政策空间。各项改革取得新成就。基本完成了187户国有企业改革，全面启动了157户厂办大集体企业改革，共支付改制成本9亿多元，盘活存量资产20多亿元。扎实推进了以免征农业税为重点的农村综合配套改革。市直事业单位人事制度改革基本完成。政府收支分类改革全面铺开，非税收入管理体制改革、部门预算和国库集中收付改革走在全省前列。

　　对外开放取得新成就。实施 “走出去、请进来”战略，积极参加省里组织的赴苏沪浙、京津鲁、闽粤、日韩俄、香港及“东博会”等经贸活动，自行组织了赴新加坡、韩国、香港及沈阳、大连、昆山等国家和地区的招商活动，成功举办了第二、三、四届中国.白山国际矿泉节和第21届长白山人参节，引进了中国电力投资公司、江苏雨润、天津康师傅、香港鼎元、修正药业、大地远通等一批战略投资者。预计2007年实际引进市外资金80亿元，比2004年翻了两番多；实际利用外资4000万美元，比2004年翻了近两番；完成外贸进出口总额2亿美元，比2004年翻了一番多。要素平台建设取得新成就。

　　实行了一系列改善金融生态环境的政策措施，金融机构对地方的支持力度进一步加大。市及5个县（市）区成立了中小企业担保公司，其中市中小企业担保公司资本金达到1.1亿元，累计担保总额近20亿元。完成了惠达投资管理有限责任公司改组。市城市信用联社改制为股份有限公司，累计向中小企业发放贷款60多亿元。开展了广泛的市校合作，20多项技术难题得到破解。中小企业孵化器和科技企业孵化器建设迈出实质性步伐。国家饮用水产品质量监督检验中心落户白山。创全国名牌产品4个、中国驰名商标3个、国家免检产品5个、地理标志保护产品7个，吉林省名牌产品17个、著名商标34个，居全省前列。

　　城乡面貌日新月异，三年发生了“三个新变化”。基础设施建设力度逐年加大，区域环境明显改善。交通条件发生新变化。完成交通建设投资40亿元，新增公路里程1100公里。宇辉铁路、鹤大线沿江至万良公路、沈长线白山至临江老岭段、长白山西坡和南坡旅游公路等重点项目相继建成通车，长白至三道沟边防公路全线贯通。在县县通油路的基础上，实现了乡乡通油路。营城子至松江河高速公路、长白山机场、环长白山旅游公路、市区南北连接线等重点工程相继开工建设。沈阳（丹东）经白山至延边和长春至白山高速公路及靖宇至松江河铁路等项目前期工作取得实质性进展。中心城区发生新变化。

　　修编了《城市总体规划》，城市布局更为科学合理。江源恢复市辖区体制，市区总面积扩大到2736平方公里，人口增加到59万人，达到中等城市规模。三年来，市区共完成城市建设总投资54.6亿元，超过前15年投资额的总和。完成了市区6个出口道路工程及星泰大桥、通江桥等5座大桥的新建或改造，通江路、靖宇路公铁立交桥和向江大桥开工建设，实施了浑江干流清淤、市区垃圾处理场、净水厂扩建、管道燃气、供水管网改造、集中供热三期、碱厂沟河和月牙河内涝治理、儿童公园综合改造、3座拦江蓄水坝、189条街（巷）路、255座公厕、12400组路灯等惠民工程，市区人均公共绿地面积达到8.3平方米，城区面貌焕然一新，成功迈进省级卫生城和省级园林城行列。新农村建设发生新变化。完成了全市156个试点村、13个试点镇的新农村建设规划。公共财政向农村的投入逐年加大，落实项目532个，投入资金6.4亿元，其中省、市帮扶部门投资2.5亿元。农业产业化进程明显加快，三年新增国家级龙头企业1户、省级龙头企业17户。农村基础设施和卫生环境有了明显改善，村容村貌大为改观，试点村安全饮水率、通电率、道路硬化率达到100%。

　　社会事业全面进步，三年实现了“三个新提高”。坚持物质文明与精神文明并重、经济建设与社会建设共举，各项社会事业取得了明显进步。社会事业建设有了新提高。83个科技项目列入国家、省科技发展计划，申报专利152项。全面落实农村义务教育“两免一补”政策，完善了扶困助学体系，改造、新建校舍17万平方米，基本完成长白山职业技术学院筹建工作。完成了长白山满族文化博物馆、市中心医院、通化矿业集团总医院住院大楼、市紧急救援中心、全民健身活动中心、白山地震台、社区用房建设以及265个自然村屯的有线电视“村村通”工程。农村新型合作医疗和社区卫生服务中心（站）建设全面铺开。

　　在第十五届省运会上金牌总数和团体总分双列第三名，连续两届蝉联全省社区体育健身大会团体总分第一名。开展了“三结合”、“五关爱”、“生殖健康家园”等活动，兑现了农村计划生育家庭奖励扶助和城镇独生子女父母退休后奖励政策，人口自然增长率控制在2.74‰以内。

　　妇女儿童发展规划稳步实施，代表全省接受了国务院妇儿工委评估。依法加强对宗教事务的管理。长白县荣获“全国民族团结进步模范自治县”。群众文明素质有了新提高。大力弘扬“向前向上，求富求强”的白山精神，全面贯彻《公民道德建设实施纲要》，深入开展“树新风正气，促和谐发展”主题教育活动和一系列以干部群众文明养成教育为主的精神文明创建活动，市民的文明程度不断提升，涌现出一批省、市级精神文明建设先进单位和个人。边防民警杨春明当选“2006年度感动吉林十大人物”。

　　维护社会稳定工作有了新提高。加大了矛盾纠纷排查化解力度，推广八道江区组建群众工作部的试点经验，信访局势保持稳定。通过“邻里守望”、“天网工程”建设、“网格化”治安巡逻等措施，加强了治安防控体系建设，加大了对法轮功等邪教组织的打击力度，组织开展了打黑除恶、命案攻坚、打击“两抢一盗”等专项行动，刑事案件发案率明显下降，人民群众安全感普遍增强。加大了对重大危险源、事故隐患和食品、药品的安全监管力度，交通、火灾事故和损失程度全省最低，实现了连续28年无重大森林火灾。强化了应急预案完善和应急救援演练，应急管理能力不断提高。加强了边境管控，爱民固边走在全省前列。

　　人民生活明显改善，三年跃上了“四个新台阶”。始终把群众利益放在心上、抓在手上，不断完善就业再就业、社会保障和社会救助体系，人民群众得到了更多的实惠。城乡居民收入跃上新台阶。2007年，预计全市城镇在岗职工平均工资1.5万元，比2004年增长57%；市区城镇居民人均可支配收入1.1万元，增长56.9%；农民人均纯收入4175元，增长35.9%。实现社会消费品零售总额77亿元，增长57.4%。人均储蓄存款1.2万元。

　　居民居住条件跃上新台阶。三年来，市区完成房地产固定资产投资45. 5亿元，是前15年投资额的总和。城市棚户区改造完成投资34.2亿元，竣工228.2万平方米，安置回迁28130户。煤矿棚户区改造完成投资5.3亿元，24栋住宅楼开工建设。采煤沉陷区治理工程开工建设53万平方米，安置居民7835户。归集住房公积金4亿元，为3000多名职工发放贷款1.8亿元。市区人均居住面积达到20.8平方米。

　　就业和再就业工作跃上新台阶。开展了全民创业促就业活动，实施了税费减免、小额担保贷款等一系列扶持政策，被列入“全国创业培训百个试点市”，培训下岗失业人员4万人，在全省率先成立了“外来务工人员服务中心”，建立了“12333”劳动保障热线。三年来城镇新增就业11.2万人，下岗失业人员再就业7.2万人，劳务输出13.1万人，农村劳动力就地就近转移4.6万人，在全省率先实现“零就业家庭”动态为零目标，城镇登记失业率控制在4%以内。

　　社会保障和社会救助工作跃上新台阶。筹措资金5.1亿元，完成了82410名国有企业下岗职工社保并轨工作。全市参加养老保险达到19.3万人，其中非公参保人员比重由2004年的17%增加到67%，发放离退休人员养老金18.6亿元，月人均标准增加219元。参加失业保险13.4万人，发放失业金2700万元，月标准提高到163.8元。启动了城镇居民医疗保险，对特殊困难群体实施财政补助5000多万元。医疗保险、工伤保险、生育保险参保人数分别达到44.1万人、12.5万人和10万人。城镇低保实现了动态管理下的应保尽保，共为18.9万人发放低保金4.8亿元；4.1万名农村低保对象救助标准从2005年的180元增加到360元。三年来用于助困、助残、助学、助医、助孤的资金4500多万元。

　　政府工作水平不断提高，三年实现了“三个新加强”。全面加强政府机关建设，政府管理水平、工作效率和服务能力明显提升。民主法制工作得到加强。自觉接受人大的法律监督、工作监督和政协的民主监督，三年共办复人大代表议案和政协委员提案413件，办结率达到100%。“四五”普法顺利完成，“五五”普法全面启动。法律服务和法律援助工作逐年加村民自治和城镇居民社区自治制度得到巩固和提高。

　　服务型、效能型政府建设得到加强。积极落实扩权强县等各项政策措施，推进了行政管理体制改革。以开展党员先进性教育为契机，各级领导班子和公务员队伍建设取得明显成效。深入开展了“治庸、治懒、治散”专项行动和“服务型机关建设行动计划”，服务质量和行政效率得到提高。坚持言必行、行必果，政府信用和为基层、为企业、为群众排忧解难的能力得到加强。强化行政执法、行政复议和错案追究制度，加强审计监督，政府法治化进程明显加快。

　　软环境治理工作得到加强。进一步清理行政许可、非行政许可项目和行政事业性、经营服务性收费，对政务大厅进行了改造。实行了“一站式”服务等十几项制度，开展了民主评议软环境和政行风等活动，加大了涉软案件的查处力度，经济发展软环境明显改善。

　　三年来，统计、环保、水文、气象、人防、价格监督、民兵预备役、外事、地方志、档案、老龄、残联等工作都为全市经济社会发展做出了积极的贡献。

　　各位代表，三年来白山的变化令人瞩目，白山的发展令人振奋。这些成绩的取得，是市委正确领导的结果，是市人大、市政协和社会各界有效监督与鼎力支持的结果，是全市各族人民群策群力、拼搏实干的结果。我代表市政府，向全市各族人民，向驻白山解放军指战员、武警部队官兵和公安司法干警，向海内外所有关心支持白山发展的各界人士，表示崇高的敬意和衷心的感谢！

　　在看到成绩的同时，我们也清醒地认识到，政府工作还存在一些不容忽视的问题，主要是：经济总量偏小，牵动产业升级的大项目偏少，经济发展方式粗放，自主创新能力不强，经济结构调整、产业优化升级、发展接续产业任重而道远；交通和城市建设历史欠账较多，基础设施投入不足，建设和改造任务繁重；社会保障能力不强，就业再就业任务很重，部分群众生活仍然比较困难；政府职能需要进一步转变，社会管理和公共服务能力有待进一步增强，发展环境亟需进一步优化。对这些问题，我们将以务实的态度、改革的精神和创新的举措，认真加以解决。

　　二、展望未来五年，全力打造精品新白山

　　未来五年，是全面实施“十一五”规划、夯实“十二五”发展基础的五年。随着经济全球化和区域经济一体化深入发展，国家支持资源型城市经济转型政策深入实施，省委、省政府加快推进能源、新型建材、长白山旅游、矿泉水、人参等新兴产业基地建设，白山面临难得的发展机遇。我市经过多年的改革发展，经济和社会各项事业全面跃升，发展活力日趋增强，全市上下政通人和求发展、为民造福奔小康的局面已经形成。同时，面对区域竞争更加激烈的新形势，我们必须切实增强忧患意识、赶超意识，在新一轮发展中抢占先机，赢得主动，努力实现在东北东部同类城市中率先崛起。

　　根据市五次党代会的部署，今后五年政府工作的总体要求是：以邓小平理论和“三个代表”重要思想为指导，深入贯彻落实党的十七大精神，坚持以科学发展观为统领，以富民强市为主题，以改革开放和自主创新为动力，切实转变经济发展方式，迅速扩充总量，加快推进工业化、信息化、城镇化、市场化和国际化进程，加强能源资源节约和生态环境保护，繁荣发展社会各项事业，不断提高人民生活水平，为早日建成工业强市、旅游大市、特产名城、宜居佳地的全面小康的长白山第一市而努力奋斗。

　　未来5年，经济发展的主要目标是，努力实现“四个翻番、三个突破”。 到2012年，全市GDP超过700亿元，完成全社会固定资产投资600亿元，地方级财政收入36亿元，全口径财政收入60亿元，年均分别增长25%以上，实现前三年翻一番、五年翻一番半的目标；规模以上工业增加值突破300亿元，人均GDP突破5.4万元，人均财政支出突破1万元，发展速度高于全省平均水平，人均各项指标保持全省领先。

　　建设实力新白山。坚持年年都是“项目建设推进年”，突出工业强市，做大特色产业，加快发展现代服务业，促进总量扩大、结构优化、集群发展。加强软实力建设，提升城市核心竞争力。到2012年，全市工业总产值突破1000亿元，年均增长29%，三次产业比重调整到9:52:39。

　　全力打造四个产值各超百亿元的主导产业集群。一是打造以矿泉饮品为主的绿色食品产业集群。矿泉饮品产能达到600万吨以上，其中高端水占矿泉水产量的35%、占产值的50%。二是打造以硅、镁、钴为主的矿产新材料产业集群。工业硅向太阳能级多晶硅和单晶硅产品延伸。金属镁向镁合金压铸件、镁合金板、超微细镁粉等下游产品延伸。推进钴矿采选冶一体化，并向钴粉、钴盐等产品延伸。三是打造以木制品加工、造纸为主的制造业产业集群。复合地板、人造板、造纸生产能力分别达到2000万平方米、60万立方米、80万吨，并向高档家具、新型装饰材料等产品延伸。加快发展汽车配件制造业。四是打造以煤电为主的能源产业集群。煤炭年产量稳定在1000万吨以上，延伸产业链条，实现原煤就地转化增值。火电总装机达到 160万千瓦，水电总装机达到 125万千瓦。靖宇核电站力争列入国家内陆核电站建设规划。

　　　　积极培育四个产值（收入）各超50亿元的优势产业集群。一是培育参药产业集群。与前沿的高新技术对接，开发生物制药、现代中医药和健康、美容系列产品。扶持天风制药等一批高成长性的企业做大做强，引进实力雄厚的大集团对参药企业联合重组，提升产业规模和层次。二是培育硅藻土产业集群。进一步整合资源，成立国际级研发中心，加快硅藻土高附加值产品的开发，拓展应用领域，快速形成产业规模。三是培育新型建材产业集群。实行与大企业、大集团联合开发，重点开发利用金属矿山尾矿、煤矸石、粉煤灰等新型建材。四是培育旅游产业集群。创建中国优秀旅游城，建设“三大旅游板块”，打造精品旅游线路，建设精品景区。开发旅游纪念品市场。加快以交通和星级宾馆为重点的基础设施建设，完善配套功能，发展会展经济，提升旅游产业层次和服务水平。

　　建设七大特色产业园区。加快新型产业基地建设，实行集约发展，重点抓好矿泉水、人参、硅藻土、林产品和硅镁铝、新型建材、汽车零配件等特色园区建设，举全市之力把矿泉水、硅藻土、人参、复合地板园区打造成世界顶级产业园区。到2012年，开发区、工业集中区实现生产总值占全市GDP的40%以上，成为引领区域经济发展的“火车头”。

　　加快发展现代服务业。重点发展旅游、金融、商贸流通、物流及信息、中介服务等现代服务业，推进体制、机制和管理创新，引进先进服务理念和行业标准，实现全面跨越。到2012年，第三产业增加值突破280亿元，年均增长31%。

　　实施“百强企业”振兴计划。集中力量培育一批具有较强支撑能力的骨干企业，到2012年销售收入1?5亿元的企业达到50户以上，5?10亿元的达到30户以上，10亿元以上的达到20户以上，并积极培育一批优良企业上市融资。

　　建设秀美新白山。坚持扩张规模与提升品位并重、加快建设与强化管理并举，优化城市空间布局，彰显城市特色，提高承载能力，增强辐射效应，建成国家园林城、国家卫生城，基本实现宜居佳地目标。

　　高起点规划。遵循“扩大规模、阶梯推进、拓展骨架、塑造特色、建设精品”的理念，着力构建“一带、两区、四组团”的中心城区空间架构，形成整合互动、功能互补、有机发展的新城区，打造具有核心竞争力的吉林东部中心城市。

　　高标准建设。坚持加快建设与彰显特色并举，提升城市品位。建成4个城区与高速公路连接出口和3条城市环路，新建7座公铁立交桥、8座跨江桥、20座步行桥、改造新建79条主次街路，形成平面街路纵横交错、立交与平面相衔接的城市交通骨架。塑造标志性建筑，新建12个广场、9个城市公园。改造治理主城区“一江八河”，建成浑江滨江景观带。打造山水秀丽、生态优美、特色鲜明的山水园林城市。统筹松江河区域规划建设，形成城市规模。集中力量抓好一批重点中心镇建设，逐步形成区域中心城市、县域中等城市、小城镇布局合理、相互促进的城镇化新格局。

　　举市兴交通。完成长春至白山、长春至松江河、沈阳（丹东）至延边白山段三条高速公路，筹建高速公路客运中心站。建成靖宇至松江河铁路，实现与东北东部铁路纵横交汇。全线贯通环长白山旅游公路、沿鸭绿江边防公路。建设桦树至漫江公路，实现环山与沿江边防公路连接。完成县乡公路建设，实现村村通油路和县城所在地、重点乡镇公路干线绕越。开通白山长白山机场国际航班，积极争取松江河至长白高等级公路、铁路和白山至临江高等级公路开工建设，形成南北连通、东西贯通、内外畅通，集航空、铁路、高速公路为一体的立体交通体系，成为东北东部重要交通枢纽。

　　建设活力新白山。大力弘扬“向前向上，求富求强”的白山精神，全方位扩大开放，吸引世界目光，使白山成为资本融入、人才聚集、投资创业的热土。

　　深化改革激发活力。建立现代企业制度，完善法人治理结构。积极推进行政管理体制改革，提高政府公共服务水平。完善政府采购制度，扩大采购范围和规模。推进部门预算和国库集中收付，完善财政管理体制。推进投融资体制改革，完善市县两级中小企业融资和信用担保体系，引进域外银行和风险投资基金，营造良好的金融生态环境。深入推进农村综合改革，加快建立农村工作新机制。

　　扩大开放增强活力。进一步扩大开放领域，优化开放结构，提高开放质量，实现由招商向选商的实质性转变，积极构建全方位、深层次的对外开放新格局。5年累计引进域外资金760亿元，实际利用外资4亿美元，完成外贸进出口总额17亿美元，年均分别增长30%。

　　自主创新催生活力。以企业为主体，以产学研相结合为途径，以健全多元化科技投入机制为保障，以提升产业竞争力为核心，推进自主创新，不断提高原始创新、集成创新和引进消化吸收再创新能力，加速科技成果向现实生产力转化。实施人才强市战略，创新人才培养、引进机制，为经济社会发展提供人才支撑和智力支持。

　　优化环境增添活力。加快培育要素市场和商品市场，维护市场秩序和信用，建立开放、有序的市场竞争环境。为经济发展创造良好的软环境，为投资者提供高效优质服务。大力推进全民创业，激发民间资本的创业活力，拓宽投资领域，培育一批主业突出、管理先进、竞争力强的民营企业。到2012年，全市民营企业突破4000户。

　　建设生态新白山。高度重视生态文明建设，加大对环境保护的投入，正确处理人与自然、发展与保护的关系，努力建设资源节约型和环境友好型社会，使白山成为东北亚地区重要的生态屏障。

　　着力优化生态环境。进一步强化森林保护和培育，继续实施天然林保护工程和退耕还林，封山育林45万亩、人工冠下补植造林30万亩、中幼林抚育70万亩，提高森林质量，生态功能显著增强。加强矿泉水富集地等生态敏感区的保护，有计划地实施生态移居工程。力争将靖宇矿泉水自然保护区列为国家级保护区。实施小流域综合治理，抓好矿山植被破坏地的生态恢复。

　　大力发展循环经济。按照“减量化、再利用、资源化”的原则，加快建立节能节水、资源综合利用的长效机制，推行清洁生产，重点抓好循环经济示范园区和重点企业循环经济试点建设。城市工业用水重复利用率达到75%以上，工业固体废弃物综合利用率达到65%以上。

　　全力改善环境质量。深入推进环境综合整治，严格控制污染物排放，主要污染物排放总量每年削减2%以上。工业粉尘和二氧化硫排放达标率达到95%以上，城区工业企业烟尘排放100%达标，市中心区空气质量国家二级标准年均达到330天；生活垃圾、医疗垃圾等有毒有害废物处置率达到100%，城镇生活污水处理达标率80%以上，工业废水处理达标率90%以上，主要江河水质达到Ⅱ类水体标准，城镇饮用水源地水质100%达标。

　　建设和谐新白山。坚持以人为本，加大公益事业投入，新增财力的70%用于改善民生，完善公共保障体系，促进城乡协调发展，人民生活更加殷实，家庭财产显著增加。

　　加快推进城乡统筹发展。建立以工促农、以城带乡长效机制，形成城乡经济社会发展一体化新格局。大力发展特色高效农业，推进农业产业化经营，增加农民收入。加快新农村建设，初步实现农村道路硬质化、通讯便捷化、饮水安全化、庭院整洁化、村庄秀美化。

　　努力提升百姓幸福指数。到2012年，城镇居民人均可支配收入突破2万元，农民人均纯收入突破7000元，低收入者收入实现翻番，人均居住面积和万人私人轿车拥有量达到全国平均水平。累计创造城镇就业岗位15万个、新增城镇就业13万人、劳务输出15万人，城镇保持无“零就业家庭”，基本实现充分就业。城镇基本养老保险、失业保险努力达到“应保尽保”。实现城乡最低生活保障、义务教育免学杂费、贫困生资助、基本医疗保险、新型农村合作医疗、社区卫生服务全覆盖。建设经济适用住房和廉租房100万平方米，解决低收入家庭住房困难问题，消除城市无房户，让全市人民住有所居。

　　全面发展社会事业。促进城乡义务教育均衡发展，大力发展职业教育，提高长白山职业技术学院办学水平，为经济发展培养更多实用人才。完善覆盖城乡居民的公共卫生和医疗服务体系，社区卫生服务达标率达到100%，乡镇卫生院、村卫生站标准化、规范化建设达到90%以上。推进文化事业繁荣发展，丰富群众精神文化生活。全面实施数字广播电视计划。继续保持群众体育和竞技体育在全省的优势地位。提高出生人口素质，改善人口结构，自然增长率控制在3.9‰以下。

　　深入贯彻党的民族宗教政策，加快少数民族地区经济社会发展。加强精神文明建设，积极开展和谐家庭、和谐社区等群众性精神文明创建活动。强化矛盾纠纷排查调处，努力把问题解决在基层和萌芽状态。深化普法和依法治理工作。加强社会治安综合治理，努力构建打防控一体化的治安防控新格局，创建全省最平安城市。完善党政军警民合力治边机制，保持边境安全稳定。坚持不懈地抓好安全生产，确保人民生命财产安全，让人民群众安居乐业。

　　各位代表！经过五年的不懈努力，使白山综合经济实力显著提升、人民群众富裕程度显著提高、可持续发展后劲显著增强，早日实现全面小康的长白山第一市目标。

　　三、明年工作建议，努力开创各项工作新局面

　　2008年是新一届政府的起始之年，做好明年的工作事关未来五年发展。主要预期目标是：全市地区生产总值增长25%，规模以上工业增加值增长26%，财政收入增长25%，全社会固定资产投资增长30%以上，社会消费品零售总额增长18%，外贸进出口总额增长30%，城镇居民人均可支配收入、农民人均纯收入分别增长12%和11%以上，城镇登记失业率控制在4.3%以内，万元工业增加值综合能耗降低10%。

　　实现上述目标，要突出抓好七个方面的工作、切实为群众办好20件实事。

　　（一）突出项目建设，快速扩充经济总量。

　　进一步加大投资拉动力度，围绕四大主导产业集群和四大优势产业集群，建设一批技术含量高、产业关联度强的大项目，优化投资结构，提高项目建设质量和投资效益。全年完成固定资产投资260亿元，人均2万元，其中工业投资130亿元以上。建成投产东圣焦化6万吨甲醇、板庙子黄金开发、鼎元林纸一体化等34个大项目；加快推进吉林黄金饮品120万吨矿泉饮品、天浩硅藻土综合开发、氧化清洁纸浆等72个投资亿元以上续建项目；开工建设煤矸石发电、钴矿采选冶炼、硅镁下游产品开发等62个投资亿元以上项目；积极抓好靖宇核电站、浮法玻璃等30个重大项目前期推进工作。

　　围绕国家产业政策支持的重点领域，谋划生成一批带动能力强、成长性好、附加值高的大项目，充实完善项目储备，滚动实施、梯次推进。鼓励支持中省属企业上项目，提高对经济发展的贡献率。严格执行重点项目建设责任制、联席会议制度，完善重点建设项目包保和考核机制，确保重点项目建设快速推进。深入开展“地质勘探推进年”活动，设立重点资源勘探基金，引进风险投资，建立多渠道投入机制。通过矿产资源的大勘探，促进优势资源的大开发，延伸产业链条，大举向终端产品进军。

　　（二）突出提速增效，提高工业经济运行质量。

　　全面实施产业升级、企业素质提升和要素平台建设计划，大力推进工业经济提速增效。全市规模以上工业总产值突破300亿元，增加值超过110亿元，净利润实现11亿元。

　　实施扶优促强。继续推进经济要素向成长性强的优势企业倾斜，全力支持36户重点企业快速扩张，力争将天风药业、丽美坚木业、金刚水泥等骨干企业培育成销售收入超5亿元的小巨人企业。加大对众邦电子、邦田木塑等40户高成长性企业的扶持力度，促其尽快成长壮大为工业经济的骨干。鼓励支持中国建材集团、香港鼎元、修正药业等优势企业对我市相关企业进行重组，组建大企业集团，形成规模效应。全市规模以上工业企业发展到300户以上。

　　加快要素平台建设。着力改善金融生态环境，鼓励金融机构加大对地方经济发展的支持力度。加强中小企业担保体系建设，增加资本金投入，提升担保能力。积极引进风险投资，提高市惠达公司融资能力，打造更优良的融资平台。进一步加大扶持、培育企业上市工作力度，推动具备条件的优良企业尽快上市。扩大孵化基地规模，推动入孵企业成长壮大。提高企业自主创新能力，加强公共研发平台和企业技术中心建设，鼓励支持开发各种专利技术、产品，巩固并开发一批具有竞争力和区域特色的名牌产品。加大科技投入，全市完成工业技术改造投资78亿元，增长30%。全力提高工业品产销率，实现新产品产值17亿元，增长47%。

　　加快园区建设。进一步完善开发区和工业园区发展规划，加快水、电、路等基础设施建设，创新管理体制和服务手段，落实年度投资计划，鼓励市内企业实行“退二进三”，推动现有产能、招商引资项目向工业园区集中。集约和节约利用土地资源，提高土地投资强度和产出效益。矿泉水、硅藻土、人参、复合地板等特色园区要加快建设进度，启动并推进汽车零部件、新型建材、硅镁等工业园区建设。

　　加快企业能力建设。深入实施千名企业家培训计划，不断提高管理、融资、营销和研发能力。充分发挥企业家协会和域外企业家俱乐部的作用，通过组织参加各种研修班以及考察、培训等活动，全面提高企业家整体素质。切实抓好企业管理创新，建立和完善职业经理人制度。妥善解决改制企业遗留问题。积极引导企业加快建立现代企业制度，完善法人治理结构，实现产权主体多元化。

　　抓好节能减排。把节能减排指标纳入经济社会发展综合考评体系，加大节能减排技改项目建设，积极推进浑江发电公司锅炉脱硫改造、东锋有色金属还原炉改造及余热利用、通钢板石铁矿选矿改造等一批节能项目。加强对煤炭、电力、冶金、建材和造纸等高耗能、高污染行业和企业节能减排的调度和监控，实行限产限排。积极推广清洁生产和新技术应用，深度开发煤矸石、粉煤灰、金属矿山尾矿等二次资源，大力发展循环经济和节约型项目。主要工业污染物化学需氧量和二氧化硫排放量分别削减2%，力争列入国家级循环经济试点市。

　　（三）突出特色富民，扎实推进新农村建设。

　　加快发展现代特色农业。按照 “一村一品、一乡一业”和“多乡一业”的发展模式，通过贴息补助、投资参股、税收优惠、资金扶持和银企对接等措施，培育壮大人参、林蛙、食用菌、中药材等特色产业，全年建设规范化特产基地100个，人参GAP标准化生产和中药材标准化栽培面积达到55%以上。发展农特产品加工业，推进产业化经营，全市省级以上龙头企业达到28户，市级龙头企业达到40户。力争建成国家级农产品质量安全监督检验中心。抓紧注册一批农特产品商标，加强绿色产品认证，打造地道长白山农特品牌，全年新认证有机食品原料生产基地3个、无公害农产品生产基地6个、绿色有机食品12个、无公害农产品30个。大力发展以长白山特种野猪为重点的特色精品牧业，特种野猪饲养户达到300户，饲养量达到3万头，建设全国最大的特种野猪养殖加工基地。

　　加强新农村建设。实施“十、百、千、万”工程，高质量建设培育10个新农村示范镇、100个新农村示范村、1000个农民致富能人、带领1万个农户增收致富。强化农民技能培训和素质提升，全面抓好农村党员智能工程、农民培训阳光工程和农民远程教育，全市农民技能培训达到1.1万人次。积极开展“白山养猪状元”、“农民科技带头人”等各种致富能人评选活动，发挥示范带动效应。大力发展新型农村合作经济组织，规范402个农民专业合作社。加强试点镇、村建设，培育一批有产业支撑和鲜明特色的先进典型。全市90%以上的行政村道路实现硬质化，70%的村实现自来水化，基础通信实现“村村通”。

　　（四）突出发展旅游业，促进现代服务业上水平。

　　大力发展旅游产业。完成《白山市旅游产业发展总体规划》修编，组织编制长白望天鹅、鸭绿江沿线和龙山湖等重点景区控制性详细规划。加快中心城市周边景区建设。加强与长白山管委会在旅游资源保护、基础设施建设和旅游市场开发等方面的合作。深度开发旅游景点，完善景区设施，开发精品旅游线路。规范旅游市场秩序，搞好旅游环境综合整治，提高从业人员素质，提升服务质量。长白山国际大酒店等星级宾馆建成并投入使用，开工建设长白山香洲大酒店、南林大酒店等一批五星级宾馆，启动长白山新天堂旅游度假区等项目，进一步提高旅游承载能力。策划举办鸭绿江、中国松花石文化旅游节和长白山冰雪旅游节，重点抓好中国北方十省旅游交易会和国外旅游交易会、旅游展览会，积极开拓国内外市场。实现旅游业总收入15.6亿元，增长30%。积极创建中国优秀旅游城，力争列入省级验收范围。

　　大力发展现代服务业。全面实施商业网点规划，建设一批具有区域特色的城乡批发市场、特产专业市场，打造优势明显、特色鲜明、布局合理的商贸物流网络。积极推动苏州商贸城、北京恒坤家具建材综合城等项目建设，争取年内投入使用。完成三江至合兴大厦地下人防商城建设。规划建设长白山特产交易市场和生产资料交易市场。进一步创新商业运营模式，大力发展连锁经营，争取引进国美等知名商业企业，推进连锁经营从传统零售业、餐饮业向其它服务领域拓展。依托物流中心，建立和完善综合性与专业性并存的多层次物流配送体系。积极发展资产评估、行业协会、咨询等中介组织和金融、保险、信息、会展等新兴服务业，推进现代服务业加快发展。

　　（五）突出开放招商，激发经济发展活力。

　　强力推进招商引资。针对国内外产业转移和资本流动的新特点，进一步强化招商引资责任意识，充分发挥政府主导作用，强化企业主体地位，围绕优势产业发展规划，健全完善项目库，创新招商方式，推动招商引资再上新台阶。精心筹划参加省里组织的各类招商活动，重点在新加坡、韩国、香港及京津鲁、沈阳、大连、唐山、长三角等国家和地区，组织开展专题招商引资活动。以引进战略投资者为重点，加强与世界500强企业及国内行业龙头企业的信息对接，密切与外国驻华使领馆、我国驻外使领馆及相关行业商会（协会）的联系，打造海外招商引资平台。办好国际矿泉节等经贸活动，不断提高白山的对外知名度。坚持招商引资与招才引智相结合，使更多的各类优秀人才汇集白山。全市实际引进市外资金105亿元，增长31%，实际利用外资5200万美元，增长30%。

　　扎实推进对外开放。适应外贸形势变化，扩大对外投资和合作领域，积极扶持重点出口创汇企业做大做强，鼓励支持企业积极申报出口名牌，提高企业出口产品质量和国际市场竞争力。鼓励有实力的企业到俄罗斯、朝鲜等国家合作开发林木、矿产等资源，建设境外原材料基地，促进境外工程承包和扩大对外劳务输出。建设复合地板、人参、碳化硅、硅藻土等出口商品基地，增强企业出口能力。加快口岸基础设施和长白国际贸易城建设，推进边境贸易快速增长。全市完成外贸进出口总额2.6亿美元。

　　加快推进民营经济发展。深入实施民营经济腾飞推进计划，进一步落实民营经济发展相关政策，加快发展科技型、加工制造型和现代服务业等民营企业。进一步建立健全服务体系，简化和规范审批登记手续，拓宽民营企业融资渠道，引导民营企业在提高经济效益的基础上，增加积累和科技投入，尽快提升发展层次。继续落实领导干部包保责任制，完善监督考核机制，切实帮助民营企业解决问题和困难。规范政府执法行为，维护民营企业的合法权益。组织开展“白山民营企业20强”评比活动，进一步提高影响力和知名度。全市民营经济实现主营业务收入290亿元，同比增长35%，实缴税金11亿元，同比增长30%。

　　（六）突出基础设施建设，塑造山水园林城市新形象。

　　加快城市建设。围绕创建国家卫生城、国家园林城目标，快速推进宜居城市建设。启动碱厂沟河综合整治、馒头山文化公园及综合改造、浑江水上公园及周边环境改造、大通沟水上乐园建设工程，靖宇路、通江路公铁立交桥年内竣工通车。完成向江大桥、通沟矿欣路公铁立交桥、城区22条规划路和建设街、东兴街等6条旧街路拓宽改造及市区主要街路、广场等8万平方米人行步道砖改造建设工程。开工建设市合兴大厦、糖酒站、电影院等区域高层建筑。

　　完成污水处理厂敷设管网一期工程和江源区垃圾处理场建设。做好满族风情园、启典公园、翠柏广场和碱厂沟河、西郊公园及所有新建街路绿化。建设扩大长白山植物园。市区新建公园绿地65万平方米、植树100万株。认真抓好县城和重点镇建设。尽快打通八道江和江源区快捷通道，加大江源新城区基础设施建设力度，逐步缩小与主城区差距。切实强化城市管理，整合城市综合执法资源，进一步完善城市管理长效机制，实施部门、企事业单位对主要街区分片包保，推进整治陋习和城市常态保洁，为创建国家级卫生城夯实基础。

　　加快交通建设。全市公路建设投资20.4亿元，在建规模1245公里，竣工915公里。沈长线老岭至临江段、市区南北连接线、马鹿沟至八号闸边防（旅游）公路、石人至新开段建成通车。基本完成松江河至长白山机场高速公路、环长白山旅游公路路基工程，力争开工建设靖宇至松江河铁路、岗山岭至抚松高速公路，东岗至长白、三道沟至集安界公路工程。加快推进白山至长春高速公路等项目的前期工作。加快农村公路建设，完成25个行政村通油路工程，通油路行政村达到全市行政村总数的88%以上。完成14个公路客货运场站建设，进一步完善交通运输体系。快速推进长白山机场建设，确保奥运会前通航。

　　（七）突出发展社会各项事业，推进和谐白山建设。

　　加快发展各项社会事业。继续加大教育投入，完善教育结构，完成长白山职业技术学院审批和第一阶段基本建设任务，实现首批招生。深入挖掘和弘扬长白山文化，完成长白山满族文化博物馆数据库建设。评定公布首批市级非物质文化遗产名录。按照市场化运作机制，争取组建各类文艺演出队伍。加快“农家书屋”工程建设。推进社区卫生服务机构建设，社区卫生服务覆盖率达到城市人口的80%以上。大力开展新型农村合作医疗，进一步提高农民看病报销比例，农民参合率达到85%以上。

　　完成市中医院移址新建，广播电视中心大楼、体育场综合训练楼竣工并投入使用。积极发展付费数字电视、视频点播等业务，完成有线电视数字化整体转换用户5万户以上。积极举办好白山市第六届运动会。全力备战全国第十一届冬运会，力争实现夺金牌的目标。稳定低生育水平，认真抓好“生殖健康家园”建设，切实提高人民群众的生育质量。依法管理宗教事务。深入开展“兴边富民行动”，促进少数民族地区经济社会发展。大力加强精神文明建设，深入开展文明村镇、文明行业、文明单位创建活动。加大“扫黄打非”和网吧整治工作力度，进一步净化文化市场。

　　保持社会稳定。深入开展平安白山创建活动，强化社会治安综合治理，“天网工程”力争实现城镇全覆盖。加强社区和农村警务网格化治安巡逻防控，从源头上控制和预防犯罪。继续保持高压严打态势，组织开展好“打黑除恶”、“网上追逃”等专项斗争，保障人民群众生命财产安全。认真做好信访工作，集中精力处理好一批信访积案、难案。高度重视安全生产工作，重点抓好煤矿、非煤矿山、危险化学品、道路交通等领域以及医院、学校、商场等人员密集场所的安全整治，严控各类事故发生。启动国有林场管理体制和集体林权制度改革，加强林政管理，严厉打击毁林犯罪，确保全年无重大森林火灾。健全公共安全、自然灾害等社会预警体系和应急救援、社会动员机制，提高各级政府处置突发公共事件能力。总结推广“爱民固边”经验，确保边境地区安全稳定。

　　切实为人民群众办好20件实事。坚持从人民群众最关心、最直接、最现实的利益问题抓起，大力实施“民生工程”。

　　在就业再就业方面：

　　1. 对“零就业”家庭、“4050”人员、城镇有劳动能力的残疾人和农村剩余劳动力实行免费培训，所需费用由财政支付。全市培训下岗失业人员2万人、农村劳动力技能培训1万人、低保家庭适龄子女培训1500人、创业培训3000人。

　　2. 建立创业促就业成功项目500个。发放小额担保贷款2000万元。新建劳务输出基地14个。农村劳动力输出新增1万人、就地转移新增1万人。

　　3. 城镇新增就业3万人，下岗失业人员再就业1.2万人，大龄就业困难对象再就业4000人，保持“零就业家庭”动态为零。

　　4. 严格执行全市最低工资标准。农民工工资当期支付率达到100%。企业劳动合同签订率达到90%以上。

　　在社会保障方面:

　　5. 财政继续对特殊困难群体给予医疗保险补贴，城镇居民参加基本医疗保险新增10万人。建立少儿医疗保险制度，建立城镇居民大额医疗补充保险，最高支付限额提高到10万元，个人年均负担比例下降30%。启动医疗保险进社区工程。

　　6. 在应保尽保的基础上，城市低保人均月补差标准由85元提高到115元。农村特困人口全部纳入低保范围，人均年补差标准提高到400元。

　　7. 对失地农民实施就业援助，特殊困难的，提供公益性岗位和小额担保贷款，并纳入城镇居民医疗保险补助范围。启动被征地农民养老保险试点。

　　在社会救助方面：

　　8．全市城乡义务教育免学杂费3000万元。动员社会力量资助贫困学生就学。

　　9. 投资420万元，新建2800平方米、160张床位的市儿童福利院。 投资450万元，新建2760平方米、150张床位的市流浪儿童救助中心。

　　10. 建设残疾人综合服务场所3000平方米，满足市区残疾人康复、器具供应、就业服务和文体活动需要。

　　在改善居民生活条件方面:

　　11. 投资3.2亿元，全面完成城市棚户区改造工程，确保剩余拆迁户全部回迁；投资12.7亿元，完成煤矿棚户区100万平方米住宅楼及基础设施配套建设工程。

　　12. 投资1.4亿元，建设经济适用住房和廉租房8.5万平方米，解决1100户低收入家庭住房困难问题。投资1821万元，对15179户最低收入住房困难家庭发放租赁补贴。

　　13. 继续实施集中供热和管道燃气工程，新增供热面积80万平方米，新增管道燃气用户1.2万户。

　　14. 完成市净水厂扩建和矿务局水源改造工程，增加市区日供水能力5万吨。加强曲家营水源地保护，启动备用水源地建设。

　　15. 投资2000万元，完成市区50座二次供水泵站及支线管网改造，提高供水能力。

　　16. 争取“慈爱蓝天”慈善资金6000万元，新建抚松县、临江市和市区垃圾处理场。

　　17．投资1200万元，建设农村安全饮水工程36处，解决2万农民吃水难问题。

　　18. 市区街路和城乡结合部安装路灯1200盏。完成新建水冲式公厕和旱厕37座，新建垃圾中转站8座。

　　19．加强市场和物价调控，重点抓好食品药品安全监管和群众生活必需品的价格调控。新增市区100公顷蔬菜基地建设，新发展蔬菜保护地200亩、裸地菜田1400亩。

　　在安全保障方面：

　　20．扩大市区治安监控系统覆盖面，安装监控点1000个，确保一级风险部门、重点单位、繁华场所技防设施普及率达到100%。新建小区封闭率达到100%。

　　各县（市）区也要结合本地实际，确定一批为人民群众办实事项目，并认真组织实施。

　　四、加强自身建设，全面提高政府工作水平

　　各位代表！白山正处于大发展、大跨越的关键时期。历史的重任、时代的挑战、竞争的态势对新一届政府提出了更高的要求。我们要全面加强自身建设，努力建设人民满意政府。

　　坚持为民执政，建设服务政府。坚持科学发展观，把政府追求的目标和老百姓的期盼一致起来，把政府的绩效考核与老百姓的评价结合起来，把对上级负责和对群众负责统一起来，全心全意为发展服务、为基层和群众服务。强化社会管理和公共服务职能，深入推进政务公开，加快发展电子政务，建立科学有效的利益协调、诉求表达、矛盾调处和权益保障机制。提高政府及其工作人员的诚信意识、责任意识，凡政府承诺的事情，坚决兑现，绝不失信于民。高度重视人民群众来信来访，拓宽社情民意诉求渠道，切实做到重大决策依民而定、工作措施应民而生、安危冷暖为民而系。

　　坚持依法行政，建设法治政府。严格按照法定权限和程序行使权力、履行职责，依法管理经济社会事务。建立健全科学决策、民主决策制度，进一步完善社会公示、新闻发布、重大决策听证和专家咨询制度。自觉接受人大的法律和工作监督、政协的民主监督，认真办理人大代表建议和政协委员提案。积极支持工青妇、民主党派和工商联等团体开展工作。进一步完善村民自治、社区居民自治和职代会、股东会等制度，保障公民的知情权、参与权和监督权。完善行政问责制度，把行政问责与行政监察、审计监督结合起来，规范执法行为，做到有权必有责、用权受监督、违法要追究、侵权须赔偿。

　　坚持务实勤政，建设高效政府。大力弘扬重实干、求实效、雷厉风行的工作作风，坚持发展看项目、经济看效益、工作看效率，对事关全局的重点工程和重要事项，只争朝夕、全力以赴，确保干一件、成一件。倡导“深入一线工作法”，推进工作重心下移，抽出更多时间深入基层、深入群众，体察民情，解决实际问题。建立目标任务、组织领导、考评奖惩“三位一体”的责任体系，完善市、县两级领导“十个一”责任制，落实交办、督办、催办、查办制度，推行具体工作限时制、工作失误追究制，坚决制止行政不作为、乱作为和推诿扯皮现象，确保机关运转更加规范协调、政府服务更加快捷高效。

　　坚持从严治政，建设廉洁政府。按照为民、务实、清廉的要求，加强政府廉政建设。完善和健全各种制度，用制度管权、按制度办事、靠制度管人。深入推进办事公开，加大对许可项目、工程招投标、资源出让、政府采购等事项的公开力度，增强行政透明度。强化对窗口单位、关键部门和实权岗位的重点监督，坚决纠正损害群众利益的不正之风，从源头上阻止“权力寻租”问题的发生。弘扬正气、抵制歪风、毫不手软地惩治腐败，为人民掌好权、用好权，努力营造风清气正的行政环境。

　　各位代表！新的目标令人鼓舞，新的形势催人奋进，新的征程任重道远。让我们在市委的坚强领导下，团结和带领全市各族人民，以高昂的斗志、饱满的热情、超常的举措、务实的作风，同心同德，砥砺奋进，为建设全面小康的长白山第一市而努力奋斗！
